# Dr. Wallace’s Paper

**Published:** 1895-05

**Authors:** 


DR. WALLACE'S PAPER.—The JOURNAL presents this remarkable paper in this issue, together with a stenographic report of a part of the discussion on same, made by the stenographer of the Dallas *News*.

It is certainly a strong paper, evincing much learning, deep research and a high order of scholarship; and even a casual reading of the remarks made upon it, must convince any unbiased person that it was not quite exactly understood;—for, taken in the sense of an "attack upon religion," as viewed by Drs. Stout, Clopton and Carhart, Head and others, it would have been indiscreet to say the least, and, indeed, eminently impolitic in the writer to read it before the Association. This is true as to the Texas Medical Association especially. Such a paper as this is construed to be by the gentlemen mentioned, would be out of place before any medical association, still it might be read with impunity before any other State association; but in view of the fact alluded to elsewhere, that the introduction of theology into the deliberations of the Texas State Medical Association, some years ago, and its prompt and vigorous denunciation by the TEXAS MEDICAL JOURNAL as unwise, injudicious, foreign to the objects and dangerous to the peace of that body, and the feeling that followed the controversy, resulted almost in the dismemberment of the society; and in further view of the fact that the old sore has only just finished healing—such a paper as this is construed by some to be, would naturally act as a bombshell.

But, it will be seen that Dr. Wallace *distinctly disclaims* any attack upon religion, and challenges any one to point out a single line in the paper that can be so construed. Who, then, is responsible for the introduction of that dangerous subject into the discussion?

That the subject has again been brought into controversy every friend and well wisher of the Association must sincerely regret; and while we have said that we would henceforth take a back seat and let the Association run itself, without any suggestions from us as to its course or policy, we must repeat, that such subject should be strictly tabooed, forbidden, in the future; for while, what Dr. Carhart says of the Association, "endorsing Jesus Christ," may be strictly true—if put to vote—we must remember that individually every member of the Association is not a Christian;—e. g., we know, to our own knowledge, that there is at least one most worthy gentleman, whose name appears on the roll of membership, and who has contributed valuable matter to our Transactions, a very distinguished gentleman, who, by the by, perhaps, fortunately, was not present, who is an orthodox Jew; and there might be others besides the JOURNAL, disposed to inquire by what authority did Dr. Carhart undertake to declare what are, or what are not the *religious beliefs* of the Association as a *body?* And, as it is essentially a scientific organization, what matters it, whether, individually or collectively, the Association *endorses* or does not endorse, Jesus Christ? Has it come to pass that a man must give satisfactory evidence of his orthodoxy as a Christian before his contributions to science will be accepted ? It seems so.

The paper, although rejected by the publishing committee of the State Association, will live in the annals of medicine. The JOURNAL is the only place where it is now published; but it will be copied, and will be read, years from now, perhaps, with a clearer and more dispassionate conception of its spirit and intent, and when the spirit "I am holier than thou" shall cut no figure, is estimating the value of a contribution to the literature of medical science. And while it may not be pronounced—far from it,—a solution of the paradox of increase of insanity *pari passu* with enlightenment and civilization, it will be valued as showing, doubtless, one of the factors or concomitants at work in its production.

The showing of hands and declarations of faith which certain of the members thought proper to make we regret to say, would have—must have made a member of the Jewish persuasion, had he been present, feel that he had no place in that body. While a stranger would most likely have drawn false conclusions as to the nature of the meeting and its objects.

Religion and theology have no place in the deliberations of a body whose declared mission is the advancement of medical and allied science, and the promotion of fraternal good will; and the JOURNAL sincerely trusts that there will not be a repetition of this dangerous precedent. In this hope we feel sure every true friend of the Association, and well-wisher for its success, must cordially join us.

